# Risk factors for leptospirosis seropositivity in slaughterhouse workers in western Kenya

**DOI:** 10.1136/oemed-2016-103895

**Published:** 2016-12-02

**Authors:** Elizabeth Anne Jessie Cook, William Anson de Glanville, Lian Francesca Thomas, Samuel Kariuki, Barend Mark de Clare Bronsvoort, Eric Maurice Fèvre

**Affiliations:** 1 Ashworth Laboratories, Centre for Immunity, Infection and Evolution, Institute for Immunology and Infection Research, School of Biological Sciences, University of Edinburgh, Edinburgh, UK; 2 International Livestock Research Institute, Nairobi, Kenya; 3 Centre for Microbiology Research, Kenya Medical Research Institute, Nairobi, Kenya; 4 The Roslin Institute, The Royal (Dick) School of Veterinary Studies, University of Edinburgh, Midlothian, UK; 5 Royal (Dick) School of Veterinary Studies, University of Edinburgh, Roslin, UK; 6 Institute of Infection and Global Health, University of Liverpool, Neston, UK

**Keywords:** Leptospirosis, Slaughterhouse

## Abstract

**Objectives:**

Leptospirosis has been documented in slaughterhouse workers around the world. Risk factors include smoking and drinking at work, and performing tasks such as cleaning offal. This paper examined risk factors for leptospirosis seropositivity in slaughterhouse workers in western Kenya.

**Methods:**

The study was conducted between May 2011 and October 2012. Questionnaires were used to collect information from workers on demographic data, health and hygiene practices in the slaughterhouse. A commercial ELISA detected antibodies to *Leptospira* spp. in serum samples and multilevel logistic regression analysis identified factors associated with leptospirosis seropositivity.

**Results:**

A total of 737 workers from 142 slaughterhouses were recruited. The seroprevalence of antibodies to *Leptospira* spp. was 13.4% (95% CI 11.1% to 16.1%). Risk factors included: having wounds (OR 3.1; 95% CI 1.5 to 6.1); smoking (OR 1.8; 95% CI 1.1 to 2.9); eating at work (OR 2.1; 95% CI 1.2 to 3.6); cleaning the offal (OR 5.1; 95% CI 1.8 to 15.0); and having a borehole for personal water use (OR 2.3; 95% CI 1.1 to 4.7). At the slaughterhouse level, risk factors included having a roof (OR 2.6; 95% CI 1.2 to 5.6) and drawing water from a well (OR 2.2; 95% CI 1.2 to 4.0). Protective factors included working in slaughterhouses where antemortem inspection was conducted (OR 0.6; 95% CI 0.4 to 1.0) and where workers wore protective aprons (OR 0.4; 95% CI 0.2 to 0.7).

**Conclusions:**

This is the first report of leptospirosis seropositivity in slaughterhouse workers in Kenya. Potential risk factors were identified and this information can be used to educate workers regarding their disease risks and ways to prevent or reduce transmission.

**What this paper adds**

- This study is the first of its type in Kenya to investigate the risk factors for leptospirosis seropositivity in slaughterhouse workers in rural Kenya.
- Personal hygiene factors have a large influence on exposure and workers who have wounds, smoke or eat at the slaughterhouse have increased risk for leptospirosis seropositivity.
- Slaughterhouse level practices such as wearing aprons and performing antemortem inspection of animals reduces leptospirosis seropositivity in workers.
- Contaminated water sources are likely to play a role in the epidemiology of leptospirosis in this region.
- This information can be used to focus intervention programmes to improve occupational safety in slaughterhouses in Kenya and potentially East Africa.

## Background

Leptospirosis is a zoonotic disease with worldwide distribution.^1^ It is caused by bacterial pathogens in the genus *Leptospira*. There are over 200 serovars of pathogenic *Leptospira* and domestic animals are maintenance hosts for a number of serovars including: cattle (Hardjo, Pomona, Grippotyphosa); pigs (Pomona, Tarassovi, Bratislava); and sheep (Hardjo and Pomona).^2^ Leptospires are maintained asymptomatically in the kidneys of the host animals and are excreted in urine.^2^

Human infections result from exposure through broken skin or mucosal surfaces to the organism in urine from an infected animal or contaminated water or soil.^3^ ^4^ Faine *et al*^5^ described three epidemiological situations that promote the transmission of leptospirosis to people: farming in temperate climates where transmission is predominantly from infected domestic animals—cattle and pigs; tropical wet areas with a range of animal reservoirs—rodents, cattle, pigs and dogs; urban situations where rodents are the predominant reservoir.

Farmers, veterinarians, slaughterhouse workers and sewer workers are occupationally exposed to *Leptospira* spp.^6^ Slaughterhouse workers have been shown, in previous studies, to have seroprevalence values twice those of other non-risk occupations.^7–9^ The risk factors identified for leptospirosis seropositivity in slaughterhouse workers are: smoking and drinking while at work, and the role of the worker in the slaughterhouse, such as cleaning or washing the offal.^4^ ^7^ ^10^ Washing offal is to remove gross faecal contamination as these materials are sold for consumption.

The majority of human *Leptospira* infections are subclinical or mild. Persons with leptospirosis often develop fever, headache, muscle pain, anorexia, nausea, vomiting, abdominal pain, rash, conjunctivitis and hepatitis.^3^ ^6^ A small number of patients will develop Weil's disease with jaundice, renal failure and haemorrhage.^11^ The microscopic agglutination test (MAT) is currently the gold standard for serodiagnosis of leptospirosis but is complex and requires experienced operators.^2^ Alternative methods include the indirect haemagglutination assay, which has variable performance, and ELISAs, which are generally recommended as a screening tool for suspect cases.^12^ ^13^ The immunoglobulin M (IgM) ELISA has improved sensitivity and specificity over the IgG ELISA for leptospirosis at all stages of disease.^12^ Unlike other infectious diseases, the development of IgG antibodies in patients with leptospirosis is highly variable, which makes it unsuitable for use in diagnostics.^14^ IgM antibodies specific for different serovars have been shown to persist for up to 6 years.^15^

There is extremely limited published material regarding the prevalence of human leptospirosis in Kenya. The first human cases were reported in 1977,^16^ and in 2011 a study investigating acute febrile illnesses in northern Kenya reported cases of leptospirosis.^17^

This study examined slaughterhouse workers in western Kenya for serological evidence of exposure to *Leptospira* spp. and identified risk factors associated with seropositivity in this population.

## Methods

### Study site

**Figure 1:**
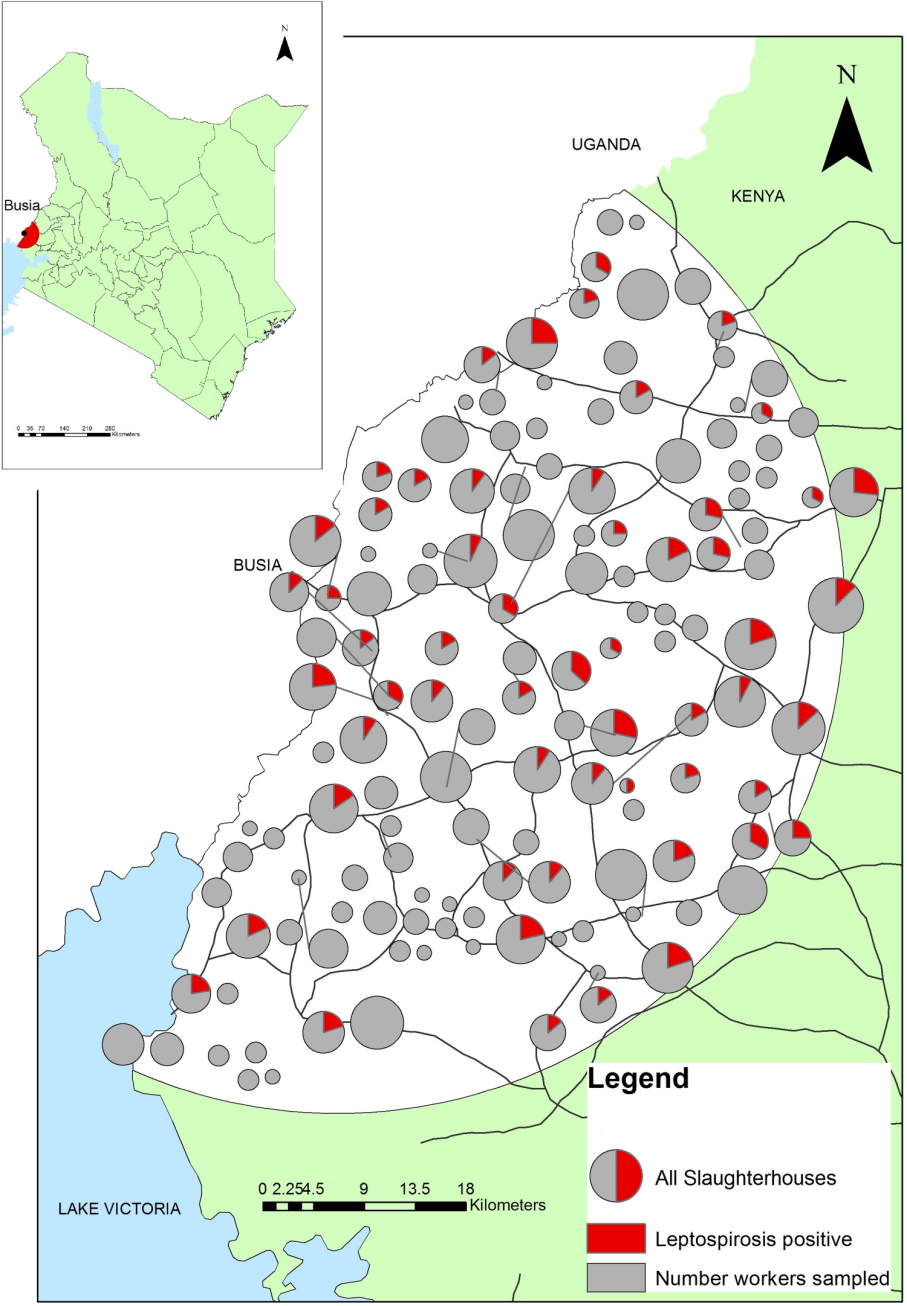
Map of study area in western Kenya demonstrating the location of the slaughterhouses. The size of the circle indicates the number of workers sampled. The red coloured wedge represents the number of leptospirosis positive workers.

The study was conducted in western Kenya in the Lake Victoria Basin region on the border with Uganda. The study area was a 45 km radius around Busia town, where the project laboratory is located (figure 1). The study area included Busia, Kakamega, Siaya and Bungoma counties. This region in the Lake Victoria crescent has one of the highest human population densities in East Africa with ∼500 people per square kilometre (estimated from the Kenyan Human Population Census of 2009). The predominant industry is mixed subsistence farming.^18^

### Study population and recruitment

A census of slaughterhouses was performed between May 2011 and January 2012. The location of slaughterhouses in the study area was obtained from the former District Veterinary Officers (now County Directors of Veterinary Services) who had oversight over meat inspection. In addition, the slaughterhouse location was requested from butchers in the market centres within the study area to ensure that no facilities were missed. Data collection was conducted between February and October 2012.

### Ethical approval

Ethical approval for the slaughterhouse study was granted by the Kenya Medical Research Institute Ethical Review Committee (SCC Protocol 2086).

### Sampling procedure

All slaughterhouses in the study area were visited 3–6 days before data collection for sensitisation and to explain the project objectives. They were assigned a unique identification number.

On the day of data collection, informed consent was obtained from all participants individually. The project enumerator explained the questionnaire and biological sampling procedures and highlighted that diagnostic tests would be performed on the samples. The participant was given a copy of the consent form to read (if literate); otherwise, the consent form was explained verbally. Participants were required to sign or apply a thumbprint to duplicate consent forms—one was retained and the other given to the participant. Inclusion criterion was all workers present at the slaughterhouse on the day of sampling, aged over 18 years. In slaughterhouses with 12 workers or less, all willing participants were recruited. In slaughterhouses with >12 workers, a random selection of 12 willing participants from the workers present on the day was sampled. This restriction was necessary due to the time required to collect data each day, and also took into account that the slaughterhouses were professional environments where income was earned by workers related to time worked. On the day of sampling, workers were assigned a number. This was written on a piece of paper and placed in a container. Numbers were selected from the container until 12 participants were chosen.

Exclusion criteria included third trimester pregnancy, severe anaemia assessed by mucous membrane pallor, being under the age of 18 years, extreme inebriation, aggression towards the project enumerators, and being aged over 85 years. All participants were offered treatment for any diagnosis made. Diagnoses were reported confidentially to participants who were treated for these conditions free of charge.

### Data collection

Four data collection tools were used to obtain data regarding slaughterhouses and workers. Interviews were conducted in Kiswahili, Dholuo, Luhya and English depending on the language in which the participant was most comfortable. Questionnaire data were recorded on a Palm operating system (Palm OS) Personal digital assistant using Pendragon Forms V.5.1 (Pendragon Software Corporation, Libertyville, Illinois, USA). Microsoft Access databases were used to manage data.

A 114-item individual questionnaire was administered to each participant by one of seven trained interviewers. Data were collected on personal history (such as age, gender, marital status and education), dietary habits, knowledge of zoonoses, risk behaviours, exposure to livestock and personal hygiene practices at the slaughterhouse. An assessment of health status for all participants was made using standard indicators including height, weight, mid-upper-arm circumference, self-reported disease episodes and a physical examination. These health indicators were recorded as part of the worker questionnaire.

A 72-item questionnaire was administered to the foreperson of the slaughterhouse regarding slaughterhouse structure, equipment and practices. The interviewer also recorded observations regarding practices where slaughtering was observed at the time of interview.

### Sampling procedure

Samples were collected from every participant who gave informed consent. Blood (14 mL) was collected by a clinical officer from each participant (10 mL plain BD Vacutainer and 4 mL Ethylenediaminetetraacetic acid BD Vacutainer) using a 21G or 23G BD Vacutainer SafetylokTM blood collection set.

### Laboratory analysis

The Panbio Leptospira IgM ELISA (Alere, Sinnamon Park, Australia) was used to screen the sera for antibodies to *Leptospira.*^19^ The ELISA is a qualitative test for antibodies to a broad range of *Leptospira interrogans* serovars including: Hardjo, Pomona, Copenhageni, Australis, Madanesis, Kremastos, Nokolaevo, Celledoni, Canicola, Grippotyphosa, Szwajizak, Djasiman and Tarassovi.^19^ An IgM ELISA was preferred to an IgG ELISA because of improved sensitivity and specificity.^12^ The ELISA was conducted as per the manufacturer's instructions as previously described.^19^ ^20^

### Statistical analysis

Questionnaire data and laboratory results were entered into Microsoft Access 2007 databases. Statistical analysis was performed in R statistical software environment (http://CRAN.R-project.org/).

The apparent prevalence estimates and their 95% CIs were calculated using the ‘epi.prev’ function in the ‘EpiR’ package (M Stevenson. epiR: An R package for the analysis of epidemiological data. In: T Nunes, C Heuer, J Marshall, *et al*. eds. R package version 0.9-57, 2014) of R (R Core Team. R: A language and environment for statistical computing. Vienna, Austria: R Foundation for Statistical Computing, 2013). Design-based adjustment was implemented using the ‘svydesign’ procedure in the ‘Survey’ package (T Lumley. survey: analysis of complex survey samples. R package version 3.28*-*2. 2012) in R. Sampling weights were calculated by dividing the total number of workers working in the slaughterhouse by the number sampled with slaughterhouse used as the clustering variable. The true prevalence estimate accounting for the test sensitivity and specificity was calculated using the ‘truePrev’ function in the ‘prevalence’ package (B Devleesschauwer, P Torgerson, J Charlier, *et al* prevalence: Tools for prevalence assessment studies R package version 0.1.0, 2013) of R. The manufacturer's recommended sensitivity (96.5%) and specificity (98.5%) for the ELISA were used. These values were chosen as there were not any published studies in the region that gave a better approximation.

### Spatial analysis

Slaughterhouses were georeferenced using a handheld Global Positioning System device (Garmin eTrex). The locations of slaughterhouses were mapped using ArcGIS V.9.1 and V.10.2.2 (ESRI, Redlands, California, USA). Base layers were provided by the ILRI geographical information systems unit (http://www.ilri.org/gis).

For mapping purposes, slaughterhouses were considered positive if one or more workers were positive for leptospirosis. A kernel smoothing approach was used to assess the density of positive slaughterhouses using the ‘sparr’ package^21^ in R. A kernel was created around each point with a fixed radius (bandwidth) of 5 km with correction for edge effects. The kernel density of seropositive slaughterhouses was divided by the kernel density of all sampled slaughterhouses in the study area creating a continuous ‘risk’ surface of the ratio of the density of seropositive slaughterhouses to all slaughterhouses. This technique does not assess clustering but produces spatially smooth risk maps that allow areas with the greatest risk for seropositivity to be identified.

### Logistic regression model

Multilevel logistic regression models were used to identify risk factors for leptospirosis seropositivity in slaughterhouse workers and estimate the strength of the relationship with the outcome. Univariable logistic regression was used to screen variables against disease exposure at the individual level. Variables were included from both the individual and slaughterhouse level data. The variables used are listed in online supplementary table S1. Variables were excluded from analysis if they were strongly correlated with another variable of interest to avoid multicollinearity problems and model estimate instability. Correlation analysis for categorical variables was performed by calculating the φ coefficient of correlation in the ‘psych’ package (W Revelle. psych: Procedures for Personality and Psychological Research. R package version 1.4.4. Evanston, Illinois, USA: Northwestern University, 2014) in R. Paired variables with a φ coefficient >0.5 were considered highly correlated and the variable that generated the highest p value during univariable logistic regression analysis was excluded.

10.1136/oemed-2016-103895.supp1 supplementary table Variables for univariable logistic regression analysis identifying risk factors for leptospirosis seropositivity in slaughterhouses workers

Variables with a p value<0.2 in the univariable analysis were used to develop a multilevel logistic regression model for each exposure. A multilevel mixed effects logistic regression model was used to account for the clustering of the workers within slaughterhouses. The model was developed using ‘glmer’ function in the ‘lme4’ package (D Bates, M Maechler, B Bolker, *et al*. lme4: Linear mixed-effects models using Eigen and S4. R package version 1.0–6, 2014). A backwards-stepwise approach was used for model selection. Starting with a full model using all predictors, variables with the highest p value were dropped in a stepwise fashion. This process was repeated until the model with the lowest Akaike's second-order information criterion (AIC) was identified.

### Model diagnostics

Variance inflation factors (VIF) were calculated to check for collinearity. VIF >4 were considered a problem and the variable removed from the model. The Moran's I Index was calculated to check for spatial autocorrelation in the slaughterhouse level residuals using the ‘ape’ package in R.^22^ The Moran's I Index measures if the outcome (the slaughterhouse level residual log odds of leptospirosis seropositivity) is clustered or randomly distributed through space. A histogram of the group level residuals was made to check for normality. The Median OR (MOR) was calculated for the final model (equation 1). The MOR is a measure of the slaughterhouse level variance (V_A_) and measures the increase in risk that a slaughterhouse worker would have if moving to a slaughterhouse of higher risk.^23^ The MOR shows the extent to which the slaughterhouse determines the individual probability of a slaughterhouse worker being seropositive for leptospirosis. The intraclass correlation coefficient (ICC) was calculated for the final model to determine the proportion of the total variance (V_A_) that could be attributed to the design (equation 2).^23^ The ICC represents correlation in the probability of seropositivity at the slaughterhouse level.1
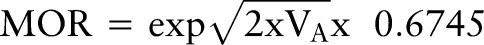
2
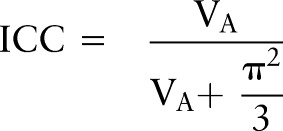


## Results

There were 156 functioning slaughterhouses in the study area between February and October 2012. Fourteen slaughterhouses refused to participate in the study. It is probable that fear of recrimination from the Department of Veterinary Services was an important factor. Seven hundred and thirty-eight slaughterhouse workers were recruited from 142 slaughterhouses, from a total of 1005 workers (73.4%). Workers were recruited from all slaughterhouse types: 274 workers in 31 mixed ruminant (cattle, goats, sheep) facilities; 292 workers in 53 cattle only facilities; and 172 workers in 58 pig only facilities. Seven hundred and thirty-seven individuals consented to giving a blood sample. Three individuals were excluded from participation after assessment by the clinical officers—one for extreme age (over 85 years) and two for severe intoxication.

The age of the participating slaughterhouse workers ranged from 18–82 years with a mean age of 39 (95% CI 39 to 40). The majority of slaughterhouse workers were men (97%; 95% CI 96% to 97%). A large number of slaughterhouse workers (82%; 95% CI 80% to 83%) had a second occupation, as butchers (42%; 95% CI 40% to 44%) or farmers (28%; 95% CI 27% to 30%). Workers had contact with livestock outside of the slaughterhouse including: poultry (88%; 95% CI 86% to 89%); cattle (72%; 95% CI 70% to 74%); goats (42%; 95% CI 40% to 44%); sheep (25%; 95% CI 24% to 27%); and pigs (37%; 95% CI 35% to 39%).

The different jobs in the slaughterhouses included slaughtermen (11%; 95% CI 9% to 14%) who were responsible for cutting the animals’ throats; flayers (75%; 95% CI 72% to 78%) who were responsible for skinning and sectioning the carcass; cleaners (4%; 95% CI 4% to 5%); the person who cleaned the offal (8%; 95% CI 6% to 10%); and foremen/owners (2%; 95% CI 1% to 3%).

The apparent prevalence of leptospirosis was 13.4% (95% CI 11.1% to 16.1%). The adjusted prevalence estimate accounting for the design effect was 13.6% (95% CI 10.9% to 16.4%). The true prevalence was determined to be 12.7% (95% CI 10.2% to 15.4%) after adjustment for the sensitivity and specificity of the test. The apparent prevalence of leptospirosis in mixed ruminant slaughterhouse workers is 13.5% (95% CI 10.0% to 18.0%); 13.4% (95% CI 9.9% to 17.7%) in cattle only slaughterhouse workers; and 13.4% (95% CI 8.0% to 18.7%) in pig slaughterhouse workers. In all cases, there was substantial overlap of 95% CIs between different slaughterhouse types, indicating no significant difference in prevalence.

**Figure 2:**
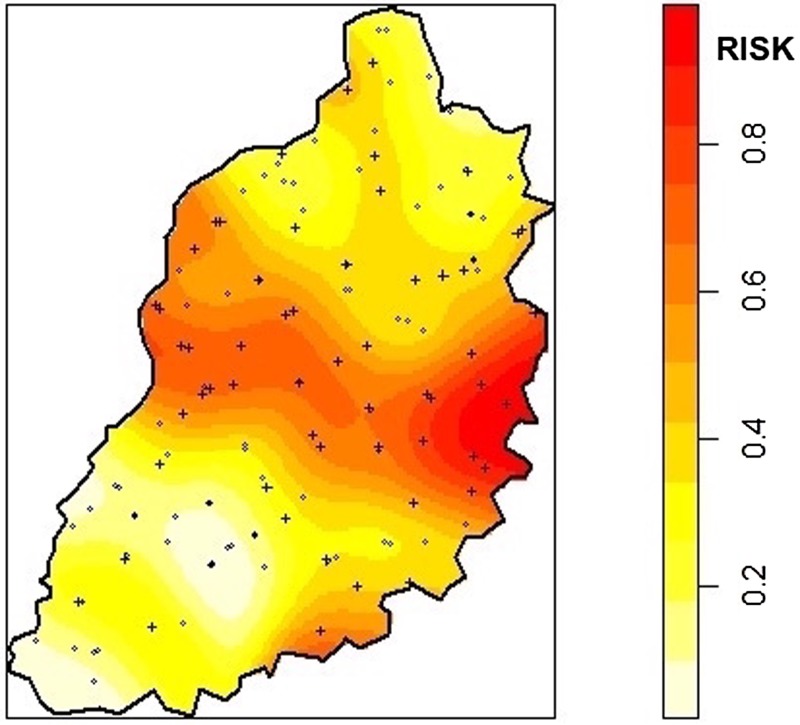
Spatially smoothed risk map for leptospirosis in slaughterhouse workers. Risk is measured as a ratio between 0 and 1 with results closest to 1 having the highest risk and demonstrated by increasing colour.

The location and proportion of leptospirosis seropositive slaughterhouse workers in each slaughterhouse is indicated in figure 1. The results of the kernel density mapping for leptospirosis in slaughterhouses are demonstrated in figure 2. Areas of greatest risk for leptospirosis seropositivity in slaughterhouse workers appear to be in the central and eastern parts of the study area.

There were no clinical signs reported within 3 months of the study that were significantly associated with leptospirosis seropositivity in slaughterhouse workers (table 1).

**Table 1:** Results of univariable analysis for clinical symptoms associated with leptospirosis seropositivity in slaughterhouse workers

Clinical symptom	Per cent (n)	Per cent of leptospirosis positive (n)	OR (95% CI)	p Value
Recent fever				
No	37.3 (275)	14.5 (40)		
Yes	62.7 (462)	12.8 (59)	0.8 (0.5 to 1.3)	0.429
Headache				
No	38.3 (282)	14.5 (41)		
Yes	61.7 (455)	12.7 (58)	0.9 (0.6 to 1.3)	0.500
Backache				
No	52.1 (384)	12.8 (49)		
Yes	47.9 (353)	14.2 (50)	1.2 (0.8 to 1.8)	0.484
Joint pain				
No	46.5 (343)	12.8 (44)		
Yes	43.5 (394)	14.0 (55)	1.1 (0.7 to 1.8)	0.632
Vomiting				
No	91.3 (673)	13.4 (90)		
Yes	8.7 (64)	14.1 (9)	1.1 (0.5 to 2.3)	0.886
Diarrhoea				
No	78.4 (578)	13.3 (77)		
Yes	21.6 (159)	13.8 (22)	1.0 (0.6 to 1.8)	0.885
Abdominal pain				
No	58.3 (430)	12.6 (54)		
Yes	41.7 (307)	14.7 (45)	1.2 (0.8 to 1.8)	0.481

### Univariable logistic regression

The complete univariable analysis for risk factors for leptospirosis seropositivity in slaughterhouse workers included 68 potential exposure variables. The individual variables that had a p value <0.2 in the univariable analysis are listed in table 2. Variables that were significantly associated with leptospirosis seropositivity in slaughterhouse workers after univariable analysis were: increasing age (OR 1.02; 95% CI 1.00 to 1.03); having wounds at the time of examination (OR 3.2; 95% CI 1.7 to 6.0); smoking at least weekly (OR 1.7; 95% CI 1.1 to 2.8); drinking alcohol (OR 1.7; 95% CI 1.0 to 2.7); cleaning the offal (OR 4.0; 95% CI 1.5 to 11.2); and eating at the slaughterhouse at any time (OR 1.7; 95% CI 1.0 to 3.0). Variables significantly associated with leptospirosis seropositivity in slaughterhouse workers after univariable analysis and that could be considered protective were: being HIV positive (OR 0.3; 95% CI 0.1 to 0.8); working at a slaughterhouse where animals were pre-examined before slaughter (OR 0.6; 95% CI 0.4 to 1.0) and working as a butcher (OR 0.5; 95% CI 0.3 to 1.0).

**Table 2:** ORs for leptospirosis seropositivity in slaughterhouse workers examining personal history variables, health, individual slaughterhouse practices and slaughterhouse factors by univariable logistic regression

Variable	Per cent of population (n)	Per cent of leptospirosis positive (n)	OR (95% CI) n=737	p Value
*Individual factors*				
Age			1.02 (1.00 to 1.03)	0.012
Other job				
None	18.5 (136)	17.6 (24)	1	
Other non-livestock	7.2 (53)	15.1 (8)	0.8 (0.3 to 2.0)	0.647
Other livestock job	4.7 (35)	17.1 (6)	1.0 (0.3 to 2.7)	0.928
Farmer	28.9 (213)	13.6 (29)	0.7 (0.4 to 1.3)	0.247
Butcher	40.7 (300)	10.7 (32)	0.5 (0.3 to 1.0)	0.034
Goat contact outside of work				
No	59.7 (440)	15.0 (66)	1	
Yes	40.3 (297)	11.1 (33)	0.7 (0.4 to 1.1)	0.133
Pig contact outside of work				
No	63.1 (465)	15.3 (71)	1	
Yes	36.9 (272)	10.3 (28)	0.6 (0.4 to 1.1)	0.055
Pigs owned				
No	70.0 (516)	14.7 (76)	1	
Yes	30.0 (221)	10.4 (23)	0.7 (0.4 to 1.1)	0.135
Private borehole* for water in the home				
No	91.9 (677)	12.9 (87)	1	
Yes	8.1 (60)	20.0 (12)	1.7 (0.9 to 3.5)	0.132
HIV				
No	87.9 (648)	14.7 (95)	1	
Yes	12.1 (89)	4.5 (4)	0.3 (0.1 to 0.8)	0.013
Have wounds at the time of examination				
No	92.4 (681)	12.0 (82)	1	
Yes	7.6 (56)	30.4 (17)	3.2 (1.7 to 6.0)	<0.001
Clinic visit in past 3 months				
No	82.5 (608)	14.5 (88)	1	
Yes	17.5 (129)	8.5 (11)	0.6 (0.3 to 1.1)	0.085
Smoke (at least once a week)				
No	76.5 (564)	11.7 (66)	1	
Yes	23.5(173)	19.1 (33)	1.7 (1.1 to 2.8)	0.024
Take alcohol (at least once a week)				
No	37.3 (275)	9.8 (27)	1	
Yes	62.7 (462)	15.6 (72)	1.7 (1.0 to 2.7)	0.040
Job in the slaughterhouse				
Slaughterman/foreman	21.4 (79)	10.1 (8)	1	
Cleaner	4.9 (36)	13.9 (5)	1.5 (0.4 to 5.1)	0.555
Cleans offal	5.7 (42)	31.0 (13)	4.0 (1.5 to 11.2)	0.008
Flayer	78.7 (580)	12.6 (73)	1.3 (0.6 to 2.8)	0.561
Wear protective aprons				
No	30.8 (227)	16.7 (38)	1	
Yes	69.2 (510)	12.0 (61)	0.6 (0.4 to 1.0)	0.071
Eat at the slaughterhouse				
No	80.5 (593)	12.1 (72)	1	
Yes	19.5 (144)	18.8 (27)	1.7 (1.0 to 3.0)	0.049
Antemortem inspection†				
No	55.8 (411)	15.8 (65)	1	
Yes	44.2 (326)	10.4 (34)	0.6 (0.4 to 1.0)	0.048
*Slaughterhouse factors*				
Number of animals slaughtered per week (increasing)			1.02 (0.00 to 1.04)‡	0.151
Roof present				
No	18.2 (134)	8.2 (11)	1	
Yes	81.8 (602)	14.6 (88)	2.0 (1.0 to 4.2)	0.061
Walls				
No	12.1 (89)	6.7 (6)	1	
Yes	87.8 (647)	12.6 (93)	2.4 (0.9 to 6.1)	0.067
Water source				
Borehole*	58.8 (433)	11.8 (51)	1	
Municipal water	16.0 (118)	11.9 (14)	1.0 (0.5 to 2.1)	0.993
Spring/well	18.0 (133)	18.0 (24)	1.8 (1.0 to 3.4)	0.063
River	7.2 (53)	18.9 (10)	1.9 (0.8 to 4.5)	0.158
Aprons worn				
No	21.7 (160)	19.4 (31)	1	
Yes	78.2 (577)	11.8 (68)	0.6 (0.3 to 0.9)	0.028

*Borehole is a deep, narrow vertical shaft in the ground used to obtain water from underground aquifers.

†Antemortem inspection is the examination of the animal before death.

‡The odds for leptospirosis seropositivity increase by 1.02 with every additional animal (cattle or pig) slaughtered each week.

Table 2 lists the variables regarding slaughterhouse level practices that had a p value <0.2 in the univariable analysis screening. After univariable analysis, working in a slaughterhouse where workers wore aprons was protective against leptospirosis seropositivity (OR 0.6; 95% CI 0.3 to 0.9).

Twenty variables from table 2 were identified for inclusion in the multilevel mixed effects logistic regression model. Variables that were obviously correlated with another variable of interest were excluded from the model. Two variables were excluded immediately for being highly correlated with another variable. Having walls in the slaughterhouse was correlated with having a roof (φ coefficient=0.76). Owning pigs was correlated with having contact with pigs outside the slaughterhouse (φ coefficient=0.69).

### Multilevel logistic regression

The final multilevel model for leptospirosis seropositivity in individual slaughterhouse workers had an AIC value of 539.25. The results of the multilevel logistic regression for leptospirosis seropositivity in slaughterhouse workers are shown in table 3. Risk factors on an individual level that were significant for leptospirosis seropositivity were: cleaning offal (OR 5.1; 95% CI 1.8 to 15.0); having a wound at interview (OR 3.1; 95% CI 1.5 to 6.1); smoking (OR 1.8; 95% CI 1.1 to 2.9); eating at the slaughterhouse (OR 2.1; 95% CI 1.2 to 3.6) and having a borehole for personal water (OR 2.3; 95% CI 1.1 to 4.7). A borehole is a deep, narrow, vertical shaft in the ground used to obtain water from underground aquifers. Individual factors that were protective against seropositivity were: being HIV positive (OR 0.3; 95% CI 0.1 to 0.9); reporting that antemortem inspection of animals was performed routinely (OR 0.6; 95% CI 0.4 to 1.0). Antemortem inspection is the examination of animals before slaughter to assess health and well-being. At the slaughterhouse level, factors that were significant for individual seropositivity include: working in a slaughterhouse with a roof (OR 2.6; 95% CI 1.2 to 5.6); and slaughterhouses that sourced water from a well or spring (OR 2.2; 95% CI 1.2 to 4.0) rather than from a borehole or river. Protective factors include working at a slaughterhouse where protective aprons were worn by workers (OR 0.4; 95% CI 0.2 to 0.7).

**Table 3:** Results of multilevel analysis for leptospirosis in slaughterhouse workers

Variable	OR (95% CI)	p Value	VIF
*Individual factors*			
Age	1.0 (0.9 to 1.0)	0.064	1.190
Job in the slaughterhouse			
Slaughterman	Ref		
Cleaner	1.3 (0.4 to 4.8)	0.677	1.534
Cleans offal	5.1 (1.8 to 15.0)	0.003	2.015
Flayer	1.4 (0.6 to 3.1)	0.468	2.470
Wounds	3.1 (1.5 to 6.1)	0.001	1.054
Smoking	1.8 (1.1 to 2.9)	0.024	1.049
Eating	2.1 (1.2 to 3.6)	0.010	1.137
HIV positive	0.3 (0.1 to 0.9)	0.031	1.035
Private borehole	2.3 (1.1 to 4.7)	0.030	1.069
Visited a clinic in past 3 months	0.5 (0.3 to 1.0)	0.065	1.041
Worker reports antemortem inspection performed	0.6 (0.4 to 1.0)	0.032	1.103
Contact with pigs outside work	0.6 (0.4 to 1.1)	0086	1.087
*Slaughterhouse level factors*			
Slaughterhouse has a roof	2.6 (1.2 to 5.6)	0.015	1.236
Water source			
Borehole	Ref		
Municipal water	1.3 (0.6 to 2.6)	0.512	1.191
Well/spring	2.2 (1.2 to 4.0)	0.007	1.172
River	1.9 (0.8 to 4.5)	0.136	1.177
Workers wear protective apron in slaughterhouse	0.4 (0.2 to 0.7)	0.001	1.242

### Model diagnostics

A number of tools were used to check the measure of fit of the model. The Moran's I Index demonstrated no evidence of residual spatial autocorrelation (value 0.007, p value 0.721). The histogram of the group level residuals had a normal distribution. The median OR for the fitted model was equal to 1; and the ICC was <1%. Both of these values indicate that, after accounting for slaughterhouse level effects in the multilevel model, little of the remaining variation in individual risk is associated with factors operating at the slaughterhouse level.

## Discussion

There was a high apparent seroprevalence (13.4% (95% CI 11.1% to 16.1%) of leptospirosis in slaughterhouse workers. Leptospirosis is commonly reported in slaughterhouse workers in many regions^24^ and a study in neighbouring Tanzania reported slaughterhouse workers to have a leptospirosis seroprevalence of 17.1% (95% CI 7.1% to 32.1%), which is similar to the findings of this study.^8^ The seroprevalence for leptospirosis in an age-matched sample from the community measured during a concurrent study was 6.5% (95% CI 5.1% to 8.3%),^25^ which is markedly lower than the prevalence in slaughterhouse workers. These data suggest that slaughterhouse workers are more at risk of exposure to leptospirosis. It may also suggest that livestock-associated serovars are prevalent in the region.

The spatial risk for leptospirosis appears to be highest through the central region of the study area, a finding that is possibly associated with cattle imported from outside the study area for slaughter; three of the main cattle markets, Ogalu, Bumala and Nambale, are located in this region. Future work will investigate the role of imported cattle and markets in the epidemiology of leptospirosis in this region.

The difference in seroprevalence between workers at the three slaughterhouse types was minimal (mixed ruminants 13.5%, cattle only 13.4% and pig only 13.4%). This suggests that the individual risk for leptospirosis seropositivity is not dependent on the type of animal slaughtered. This is in contrast to other studies that have shown an increased risk in specific slaughterhouse types, for example, sheep slaughterhouses.^10^

This study used a commercial IgM ELISA on a single serum sample to determine seropositivity. Although the MAT is considered the gold standard for leptospirosis diagnosis, the complexity of the test limits its use to reference laboratories, so commercial IgM ELISAs are used commonly in resource poor settings.^14^ ^26^ In addition, a full range of serovars is not available for this region. Sensitivity of the ELISA compared with MAT is generally good overall, although there is the possibility of regional variation and while the ELISA detects antibodies to a range *Leptospira,* it does not distinguish between serovars.^13^ ^14^ ^19^

The performance of the IgM ELISA has yet to be determined in a Kenyan setting. It is impossible to determine without reference to a ‘gold standard’ the performance of the ELISA in this region. The ELISA was developed to detect antibodies to a wide range of leptospiral antigens. However, it is possible that the ELISA may not detect the serovars common in this environment which would affect the sensitivity of this test.^20^

Unexpectedly, there were no clinical symptoms, reported within the previous 3 months, associated with leptospirosis seropositivity in slaughterhouse workers. The ELISA used in this study detects IgM antibodies which suggest recent infection; however, IgM antibodies to leptospirosis can persist for 6 years, suggesting that the majority of workers were exposed for more than 3 months prior to the study.^15^

The multilevel logistic regression analysis demonstrated a number of variables to be associated with leptospirosis seropositivity in slaughterhouse workers. Workers who cleaned the offal were at increased risk of leptospirosis seropositivity compared to workers in other positions in the slaughterhouse (OR 5.1; 95% CI 1.8 to 15.0). It has been previously reported that different roles or positions in the slaughterhouse have differing levels of risk for leptospirosis, with those who have contact with the viscera being at higher risk.^7^ ^10^ This is most likely due to contact with urine or infected organs during evisceration of the carcass. The kidney and liver are target organs for the pathogen in clinically affected animals, so contact with these organs could potentially result in infection.

Eating at the slaughterhouse (OR 2.1; 95% CI 1.2 to 3.6) and smoking (OR 1.8; 95% CI 1.1 to 2.9) were shown to be risk factors for leptospirosis seropositivity. Smoking and eating increases the possibility of transmitting leptospires from contaminated hands to the mucous membranes of the mouth.^27^ Similar findings have been reported in pig slaughterhouse workers in the USA where smoking and drinking beverages at work were reported as risk factors for leptospirosis.^4^ The same study in the USA reported that washing hands after work was protective, which was not found in this study.

Workers with wounds were more likely to be seropositive to leptospirosis (OR 3.1; 95% CI 1.5 to 6.1). This result is consistent with regular pathways of infection through cuts and abrasions.^28^ Workers in slaughterhouses where protective aprons were worn were at lower risk of testing seropositive for leptospirosis (OR 0.4; 95% CI 0.2 to 0.7). Wearing protective clothing has been shown to be protective for other zoonotic pathogens such as *Brucella* spp.^29^ Since *Leptospira* spp. are transmitted through cuts and mucous membrane contact, protective equipment that covered the hands and face would be most protective; aprons would not necessarily prevent exposure. It is possible that the use of protective clothing is a proxy for an unidentified factor such as greater care or risk aversion, as has been seen in other studies.^30^

The prevalence of HIV in the study population was 12.1% (95% CI 9.9% to 12.6%). This is similar to the findings in an age-matched sample of the community where HIV prevalence is 10.8% (95% CI 9.1% to 12.9%).^25^ In this study, people with HIV were at reduced risk of leptospirosis seropositivity (OR 0.3; 95% CI 0.1 to 0.9). This result is similar to that of a hospital-based study in Tanzania.^31^ Biggs *et al*^31^ did not offer an explanation for this finding and concluded that further investigation of coinfection in HIV and leptospirosis endemic areas was warranted. A study in India showed that mortality was high in coinfected individuals.^32^ It is possible that high morbidity and mortality in coinfected individuals explains their absence from this study group.

Workers who reported that animals were pre-examined before slaughter had reduced risk of being seropositive (OR 0.6; 95% CI 0.4 to 1.0). Animals with leptospirosis can present with fever, inappetence, mastitis, jaundice, anaemia, pneumonia or abortion. However, the vast majority will be asymptomatic and animals can shed *Leptospira* spp. in their urine for a long time after infection.^33^ These animals are unlikely to be removed from slaughter due to clinical illness. This finding might be confounded by another unidentified factor associated with better management at the slaughterhouse level.

Workers who worked in slaughterhouses that have a roof had a higher risk of leptospirosis seropositivity (OR 2.6; 95% CI 1.2 to 5.6). Leptospirosis has been shown to survive in the environment in diluted urine in direct sunlight for 2 days and in cooler-shaded environments for longer.^34^ These findings could suggest that leptospires survive longer in slaughterhouses that have a roof if they are not adequately cleaned, leading to exposure of workers.

Using well or spring water at the slaughterhouse was associated with increased leptospirosis seropositivity in slaughterhouse workers (OR 2.2; 95% CI 1.2 to 4.0). Contaminated water can be a source of infection^2^ and it is possible that wells/springs within slaughterhouses are inadvertently contaminated by slaughter waste or animal urine. Wells/springs rely on groundwater and may be contaminated as opposed to boreholes that source water from underground aquifers. Using a private borehole for personal use was also associated with leptospirosis seropositivity, which may also be the result of contaminated water sources (OR 2.3; 95% CI 1.1 to 4.7).

This study investigated a large number of occupationally related variables for exposure to leptospirosis in slaughterhouse workers. The limitations of this study are that non-occupationally related exposures were not extensively covered. Information was gathered about exposure to livestock outside of work and secondary occupational exposures and these were not significant factors in the final multivariable logistic regression model. The questionnaire was developed to capture data on a range of zoonotic disease risks specifically to do with working in slaughterhouses, so variables regarding recreational exposure to water and other risk factors related to leptospirosis exposure such as sugarcane farming, which is a common activity in the area, were not captured.^5^

The findings from this study can be used to target training programmes to reduce occupational exposure to zoonotic diseases in slaughterhouses. Education of slaughterhouse workers should focus on both sanitation in the workplace and personal hygiene, such as: effective cleaning of slaughterhouses; using personal protective equipment; covering cuts; hand washing and hygiene; and training inspectors in antemortem examination of animals.

## Conclusions

This study is the first of its type in Kenya to investigate the risk factors for leptospirosis seropositivity in slaughterhouse workers. The workers with the greatest risk of leptospirosis seropositivity are those who have contact with the viscera through cleaning the offal. This seropositivity is most likely due to their intimate contact with infected organs.

Personal hygiene factors appear to have the most influence on the risk of transmission of this zoonotic disease. Workers who have wounds, smoke and eat at the slaughterhouse have higher risk for leptospirosis seropositivity than other workers.

In order to improve conditions in slaughterhouses in western Kenya and reduce exposure of workers to zoonotic diseases, workers need to be educated regarding their disease risks and ways to prevent or reduce transmission, especially with regard to use of personal protective measures.
