# Supplementary material for: Risk factors for leptospirosis seropositivity in slaughterhouse workers in western Kenya
Source: Occup Environ Med. 2016 Dec 2;74(5):357–65. doi: 10.1136/oemed-2016-103895 (PMC5520261; doi:10.1136/oemed-2016-103895)
Supplement: supplementary table — Variables for univariable logistic regression analysis identifying risk factors for leptospirosis seropositivity in slaughterhouses workers [file oemed-2016-103895supp001.pdf]

**Individual variables**	**Individual variables - continued**
Age	Seeing rats at the slaughterhouse
Education level- none, primary secondary and above	Seeing the meat inspector at the slaughterhouse
Residing outside the study area	Antemortem inspection performed
Cattle contact/ownership	Wearing protective clothing
Sheep contact/ownership	Wearing protective boots
Goat contact/ownership	Seeing rats at the slaughterhouse
Pig contact/ownership	**Group level variables - slaughterhouses**
Dog contact	Having a roof
Hunting wildlife	Having a cement floor
Drinking cows milk	Handwashing facilities
Drinking goats milk	Running water
Eat beef/pork	Having a latrine
Drinking animal blood	Meat inspector visits
Smoking weekly	Antemortem inspection performed
Drinking alcohol weekly	Type of animals slaughtered
Name zoonoses	Cleaning with soap
Personal water source – borehole, river, municipal, well/spring	Workers wear protective clothing
Using latrine for defecation	Workers wear boots
Having a wound	Seeing dogs around the slaughterhouse
Being inebriated at slaughterhouse	Seeing rats around the slaughterhouse
Being HIV positive	Soap provided
Frequency of injury at slaughterhouse	Seeing dogs around the slaughterhouse
Visited a health clinic in previous 3 months	Seeing rats around the slaughterhouse
Length of time as slaughterhouse worker	Water source – borehole, river, municipal, well/spring
Job in the slaughterhouses slaughterman, cleaner, flayer, cleans offals	Number of people working in slaughterhouse
Job outside the slaughterhouse – butcher, farmer, non livestock work, other livestock work	Number of animals slaughtered per week
Washing hands before eating	Slaughtering sick animals
Washing hands after slaughtering	**Clinical symptoms**
Washing hands before slaughtering	Fever
Washing hands after latrine	Headache
Eating at slaughterhouse	Backache
Slaughtering sick animals	Joint pain
Using soap in slaughterhouse	Abdominal pain
Animal type slaughtered	Diarrhoea
Animal number slaughtered per week	Vomiting

Supplementary table 1: Variables for univariable logistic regression analysis identifying risk factors for leptospirosis seropositivity in slaughterhouses workers
